# Screening of Immune-Related Genes and Predicting the Immunotherapeutic Effects of Formononetin in Breast Cancer: A Bioinformatics Analysis

**DOI:** 10.1155/2022/9942373

**Published:** 2022-04-15

**Authors:** Xiaotong Song, Jie Li

**Affiliations:** Department of Oncology, Guang'anmen Hospital, China Academy of Chinese Medical Sciences, Beijing 100053, China

## Abstract

**Objective:**

Immunotherapy is a promising breast cancer treatment. Nonetheless, tumor heterogeneity and the interaction between immune cells in the tumor microenvironment limit its effectiveness. Formononetin—extracted from the Chinese medicinal plant *Astragalus membranaceus*—can inhibit tumor growth, induce apoptosis and angiogenesis, and reverse multidrug resistance. However, its efficacy and mechanism of action on the immune cells in breast cancer remain unclear. Here, we screened immune-related genes of breast cancer to determine the potential of formononetin as a therapeutic.

**Methods:**

GSE103512 and GSE139038 breast cancer microarray data and immune-related gene data were obtained from the GEO and ImmPort databases, respectively, to analyze the differentially expressed immune-related genes (IRGs) in breast cancer tissues compared with normal breast tissues. Protein-protein interaction (PPI) analysis was performed using the STRING database to screen differentially expressed IRGs based on the topological parameters. The Kaplan–Meier test was applied to detect differentially expressed IRGs associated with breast cancer survival, and the interaction of formononetin with differentially expressed IRGs was analyzed using molecular docking. Finally, the relationship between differentially expressed IRGs and breast cancer immune cell infiltration was analyzed using the TIMER2.0 database.

**Results:**

A total of 29 differentially expressed IRGs of breast cancer were screened through GEO and ImmPort databases and 10 key differentially expressed IRGs based on the topological parameters from the PPI network. Among these, CXCL12, ESR1, IGF1, and FOS were associated with breast cancer survival. Furthermore, IGF1, ESR1, and CXCL12 were found to have stable binding sites for formononetin. These genes were associated with substantial immune cell infiltration in breast cancer tissues.

**Conclusion:**

In conclusion, formononetin may exert antitumor effects by acting on CXCL12, ESR1, and IGF1 and may have a potential synergistic effect with immune checkpoint inhibitors.

## 1. Introduction

Breast cancer is the most prevalent cancer worldwide with an estimated 2.26 million new breast cancer cases in 2020, accounting for about 11.7% of the total new cancer cases [1]. Breast cancer deaths account for approximately 15% of all cancer deaths, making it one of the main causes of cancer-related deaths [2]. Multimodal breast cancer therapies have been documented. These include chemotherapy, surgery, targeted therapy, hormone replacement therapy, radiation therapy, complementary therapy, gene therapy, and stem cell therapy [3]. However, advanced and recurrent breast cancer are still difficult to cure.

In recent years, immunotherapeutic avenues have advanced the treatment of refractory cancers. The immune system, the main of defense in the human body, consists of a variety of immune factors, including cells and tissues [4], which are related to the standard treatment and long-term survival of patients [5]. Breast cancer has been considered an immunologically “cold tumor” due to the relatively low levels of T cell infiltration and mutational load [6]. Recently, the role of the immune system has been critically reevaluated in breast cancer progression and treatment response, providing an opportunity for immunotherapy. The expression of the immune checkpoint protein—programmed cell death protein 1 and its ligands (PD-1 and PD-L1)—in the tumor microenvironment supports the role of immune editing in breast cancer [7, 8]. However, owing to the significant differences in immune cell infiltration and immune response in breast cancer, many patients with PD-1+ cancers fail to show a long-pasting suppressive response to PD-1 [9, 10], and overcoming this challenge continues to be a bottleneck in cancer treatment.

Isoflavones are estrogen-like polyphenols with potent anticancer properties [11]. Isoflavones exert anticancer effects via nonhormonal mechanisms [12]. They can inhibit angiogenesis, induce cell apoptosis, inhibit DNA topoisomerase, and suppress cancer cell differentiation [13, 14]. Formononetin—an isoflavone extracted from the Chinese medicinal plant *Astragalus membranaceus*—has multiple pharmacological effects [15]. It can inhibit tumor growth, induce apoptosis, prevent angiogenesis, and act against multidrug-resistant tumors [16, 17]. In addition, formononetin can regulate various transcription factors and growth factor-mediated carcinogenic pathways. Therefore, it can not only inhibit tumor growth but also alleviate the chronic inflammation related to chemotherapy resistance [18].

Many diseases, including cancers, have complex pathogenesis and seldom respond effectively to a single treatment forever [19]. The small molecule compounds from herbal medicines have the property of multiple target interventions and great potential as drug candidates in complex and refractory diseases. However, the lack of comprehensive knowledge about the mechanism of drug action has hindered the broader application of natural products in drug research and development [20]. Bioinformatics analysis of microarray data is a new approach for exploring disease-related gene expression, predicting molecular interactions, determining key regulatory pathways, and identifying targets for disease therapy [21, 22]. Herein, we applied bioinformatics analysis and molecular docking techniques to predict the comprehensive mechanism of action of the natural compound formononetin in tumor immunity and provide a theoretical reference for immunotherapy of breast cancer.

## 2. Materials and Methods

### 2.1. Data Source and Processing

Gene Expression Omnibus database (GEO, https://www.ncbi.nlm.nih.gov/geo/) is a public functional genomics database that provides array and sequence-based data. The original RNA detection in *Homo sapiens* was searched using the keyword “breast cancer,” and GSE103512 and GSE139038 were selected. GSE103512 came from the GPL13158 (HT_HG-U133_Plus_PM Array Plate) platform and included 65 breast cancer tissues and 10 normal breast tissues (data submitted on Sep. 05, 2017). GSE139038 was obtained from the GPL27630 (Block_Column_Row ID) platform and included 41 breast cancer tissues and 24 normal breast tissues (data submitted on Oct. 17, 2019). The differentially expressed genes (DEGs) between breast cancer tissues and normal breast tissues were screened using GEO online analysis tool. DEGs with *P* < 0.05 and (logFC) > 1 were regarded significantly differentially expressed. The DEGs shared by the two datasets were obtained using Venny 2.0.2, and the heat map was generated using the R ggplot2 package. The Database for Annotation, Visualization, and Integrated Discovery (DAVID: https://david.ncifcrf.gov/) is a comprehensive functional annotation tool and is used for gene ontology (GO) and Kyoto Encyclopedia of Genes and Genomes (KEGG) pathway enrichment analysis of DEGs. The cutoff threshold was set as *P* < 0.05.

### 2.2. Differentially Expressed IRGs Analysis and PPI Network Construction

We obtained the list of IRGs from the Immunology Database and Analytical Portal (ImmPort, https://www.immport.org/) database and used Venny 2.0.2 to obtain the differentially expressed IRGs that are common between DEGs and IRGs. The STRING database (https://String-db.org/cgi/input.pl) and Cytoscape 3.5.1 software were used to construct the protein-protein interaction (PPI) network of differentially expressed IRGs in breast cancer [23, 24]. CytoNCA performs network centrality analysis to identify key genes in biological networks and includes a variety of powerful visual analysis modules to generate output in various forms, such as graphs, tables, and charts, and to analyze the associations between all measures [25]. The CytoNCA plug-in was used to analyze the topology of the PPI network. The primary nodes in the PPI network were identified using topology analysis and regarded as the primary differentially expressed IRGs that can be considered as biomarkers in breast cancer. GO and KEGG analyses explored significantly dysregulated pathways and biological processes, cellular components, and molecular functions related to differentially expressed IRGs using the DAVID database. The cutoff threshold was set as *P* < 0.05.

### 2.3. Kaplan–Meier Test

Kaplan–Meier plotter (https://kmplot.com/analysis/) was downloaded from GEO, EGA, and TCGA with gene expression data, overall survival (OS), and recurrence-free survival (RFS) information and processed using the PostgreSQL server to integrate both gene expression and clinical data [26]. It included 54,675 genes affecting individual survival in 10461 cancer samples (5143 breast cancers, 1816 ovarian cancers, 2437 lung cancers, and 1065 gastric cancers). To analyze the prognostic values of specific genes, patient samples were divided into two groups based on the expression of various quartiles of the proposed biomarkers. Univariate and multivariate Cox proportional-hazards analyses were performed by Kaplan–Meier survival plots to compare the two patient cohorts. The risk ratios were calculated with 95% confidence intervals and log-rank *P* values. OS refers to the duration from the time of diagnosis till death due to any cause. RFS refers to the time from lesion clearance to tumor recurrence. The Kaplan–Meier plotter chi-square test was used to determine the relationship between differentially expressed IRGs and breast cancer survival. The cutoff threshold was set as *P* < 0.05.

### 2.4. Molecular Docking

The 2D structure of formononetin was downloaded from the PubChem database (https://pubchem.ncbi.nlm.nih.gov). Water and ligands were removed from the protein using PyMOL 1.7.6 [27]. Protein crystal structures of differentially expressed IRGs were downloaded from the PDB (https://www.rcsb.org/) and stored in.pdb format. The minimum energy was calculated using the Chem3D Pro14 software and then saved in.mol2 format. AutoDock, a suite of automated docking tool designed to predict the binding of small molecule substances to receptors with known 3D structures, was applied to add hydrogen to the target protein and calculate Gasteiger charges. Differentially expressed IRGs (as receptors) and formononetin (as a ligand) were imported into AutoDock for molecular docking and visualized using PyMOL. The conformation with the lowest energy was regarded as the optimal candidate.

### 2.5. Correlation Analysis between Differentially Expressed IRGs and Immune Cell Infiltration in Breast Cancer

Tumor immune estimation resource (TIMER2.0) database (https://timer.cistrome.org) is used for analyzing tumor immune infiltration and visualizing the results [28]. The TIMER algorithm was used to investigate the infiltration levels of CD4+ and CD8+ T cells, B cells, dendritic cells, macrophages, and neutrophils. The relationship between differentially expressed IRGs and the level of immune infiltration in breast cancer was explored in “Gene” module. *P* < 0.05 was set as the cutoff criterion. The correlation between risk score and immune infiltration was calculated using Pearson correlation.

## 3. Results

### 3.1. Differentially Expressed Gene Analysis

**Figure 1:**
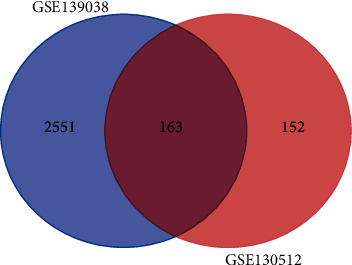
Overlapping 163 DEGs identified from two datasets. The blue area represents GSE139038. The red area represents GSE103512. The intersection means the DEGs shared by the two datasets.

**Figure 2:**
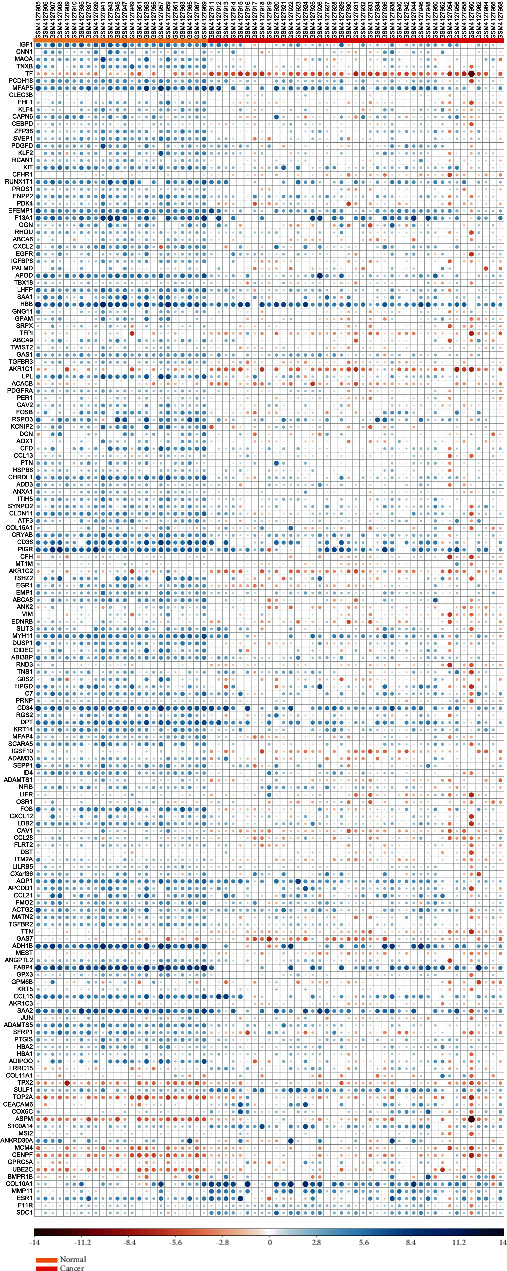
The heatmap of DEGs. The color from blue to red represents a trend from low expression to high expression.

**Figure 3:**
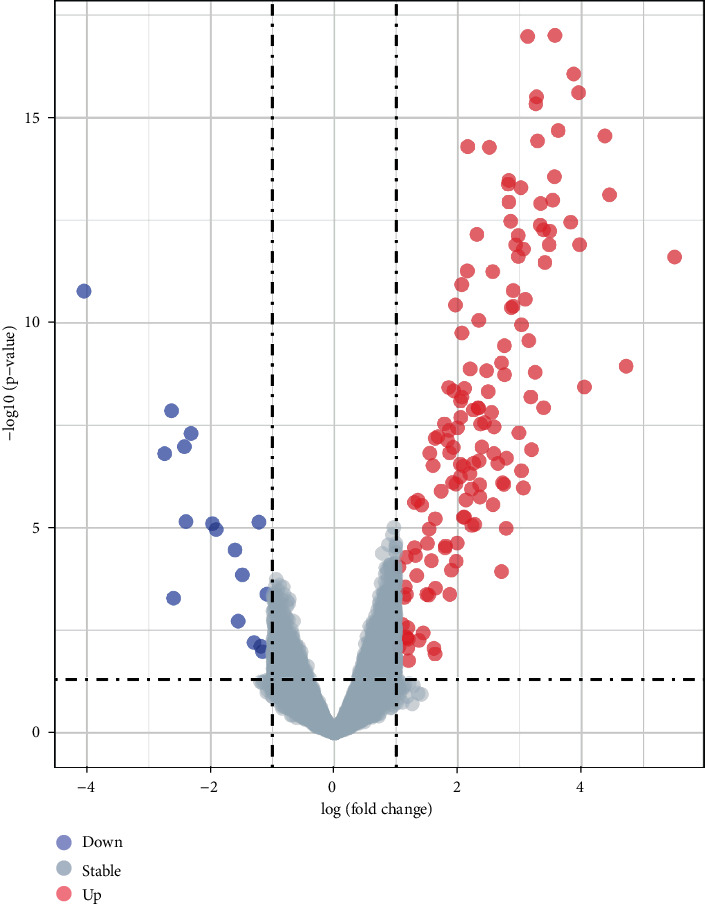
The volcano plot of DEGs. Red points represent upregulated DEGs, and blue points represent downregulated DEGs.

GSE103512 and GSE139038 were downloaded from the NCBI-GEO database. Based on GEO-GEO2R analysis, DEGs were identified by setting *P* < 0.05 and (logFC) > 1. We screened a total of 163 DEGs from GSE103512 and GSE139038 employing Venny 2.0.2 (Figure 1). Among these, 142 were upregulated and 21 were downregulated. The heatmap and volcano plot were generated using the R ggplot2 package (Figures 2 and 3).

### 3.2. Gene and Pathway Enrichment Analysis of DEGs

The DAVID database was used to enrich the GO and pathways of DEGs. We carried out GO enrichment analysis on 163 DEGs, based on categories of the molecular function, biological process, and cell components (*P* < 0.05). There were 45 terms of molecular functions, including heparin binding (GO:0008201), chemokine activity (GO:0008009), peroxidase activity (GO:0004601), transcription factor binding (GO:0008134), and chemokine receptor binding (GO:0042379); 28 terms were identified for cellular components, such as extracellular exosome (GO:0070062), proteinaceous extracellular matrix (GO:0005578), extracellular matrix (GO:0031012), membrane raft (GO:0045121), and endocytic vesicle lumen (GO:0071682); and 126 terms were identified for biological processes, including cell chemotaxis (GO:0060326), positive regulation of fibroblast proliferation (GO:0048146), cellular response to interleukin-1 (GO:0071347), and positive regulation of cell proliferation (GO:0008284). The KEGG pathway enrichment analysis was performed on 163 DEGs using the DAVID database, setting *P* < 0.05 as the cutoff value. The results gave eight pathways, including proteoglycans in cancer (hsa05205), cytokine-cytokine receptor (hsa04060), pathways in cancer (hsa05200), chemokine signaling pathway (hsa04062), and choline metabolism in cancer (hsa05231).

### 3.3. PPI Analysis of Differentially Expressed IRGs

**Figure 4:**
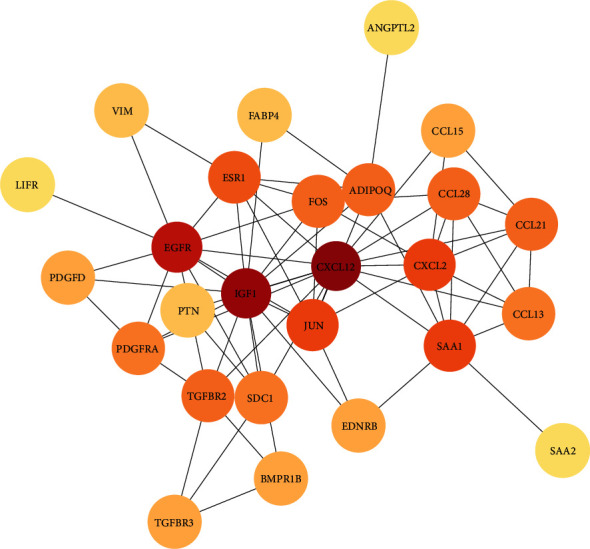
PPI protein interaction network diagram of differentially expressed IRGs. The nodes in the diagram change from yellow to red, indicating that the “degree” is larger.

Venny 2.0.2 was used to screen the genes shared by DEGs and IRGs and 29 differentially expressed IRGs were identified. We used the STRING database and Cytoscape software to construct a PPI network with 26 nodes and 69 edges and to quickly analyze the interaction of differentially expressed IRGs. Cytoscape's plug-in CytoNCA was used to perform topology analysis of the PPI network (Figure 4). According to the criteria, “BC,” “CC,” “DC,” “EC,” “LC,” “NC,” “SC,” and “IC,” the top ten candidate nodes were selected, including CXCL12, IGF1, EGFR, JUN, CXCL2, ESR1, FOS, SAA1, CCL28, and TGFBR2 (Table 1).

### 3.4. Enrichment Analysis of Differentially Expressed IRGs

**Figure 5:**
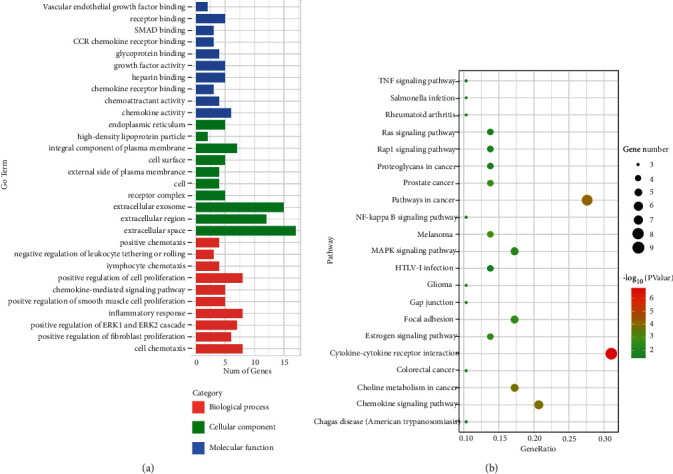
GO and KEGG functional enrichment analysis of differentially expressed IRGs. (a) Top 20 biological processes, cellular components, and molecular functions for DEGs (*P* < 0.05). (b) KEGG pathways for DEGs (*P* < 0.05).

The DAVID database was used for GO and KEGG enrichment analysis of 29 differentially expressed IRGs. Both analyses used *P* < 0.05 as the cutoff value. Enrichment analysis of these differentially expressed IRGs revealed possible pathways involved in breast cancer immunity. GO analysis revealed 21 terms for molecular function, 11 for cellular components, and 73 for biological processes (Figure 5(a)). KEGG pathway enrichment analysis of differentially expressed IRGs identified 21 pathways (Figure 5(b)) and among them were positive regulation of fibroblast proliferation, inflammatory response, positive regulation of ERK1 and ERK2 cascade, lymphocyte chemotaxis, estrogen signaling pathway, MAPK signaling pathway, NF-kappa B signaling pathway, Ras signaling pathway, and TNF signaling pathway associated with PD-1/PD-L1-mediated immune escape.

### 3.5. Survival Analysis of Differentially Expressed IRGs

**Figure 6:**
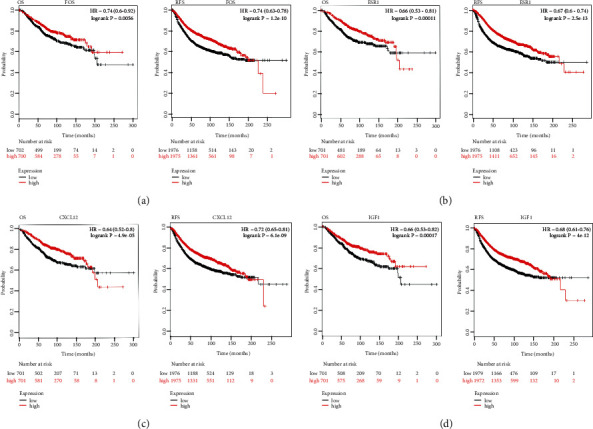
Kaplan–Meier curves of four prognostic differentially expressed IRGs in BC. (a) FOS. (b) ESR1. (c) CXCL12. (d) IGF1. The criterion is that when the *P* values of RFS and OS are <0.05, it is considered that DEGs are associated with the survival of BC.

To further elucidate whether these differentially expressed IRGs contributed to the survival period in patients with breast cancer, we analyzed OS and RFS for differentially expressed IRGs utilizing Kaplan–Meier plotter. The results showed that CXCL12, ESR1, IGF1, and FOS were significantly associated with the survival of breast cancer. In addition, we found that high expression levels of six differentially expressed IRGs were associated with poor RFS and OS in breast cancer patients, suggesting the remaining four differentially expressed IRGs can be used as biomarkers for breast cancer (Figure 6).

### 3.6. Molecular Docking Model

**Figure 7:**
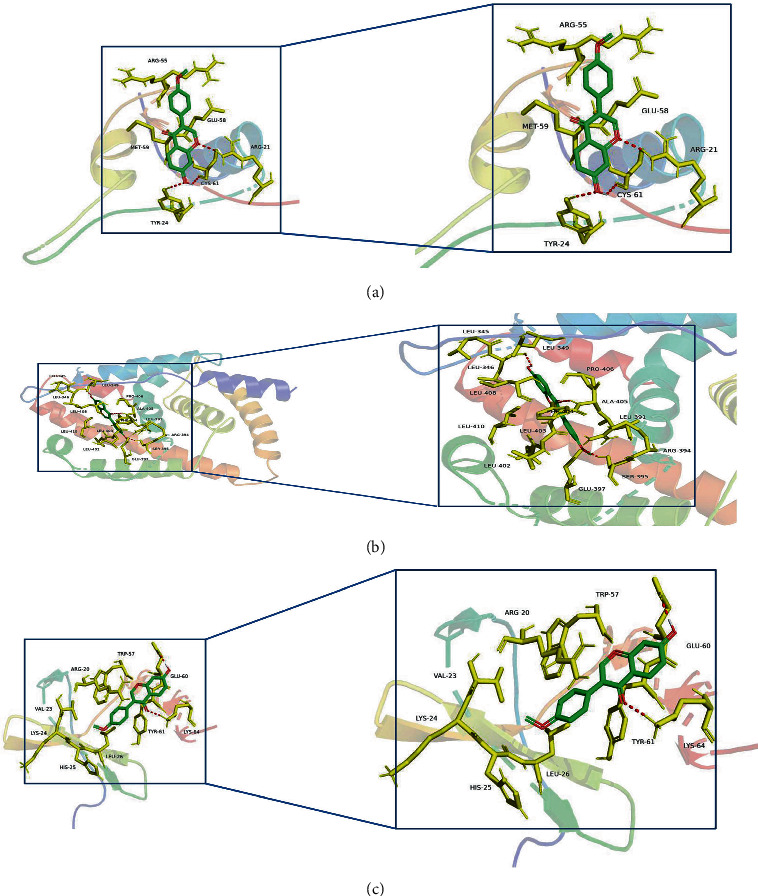
Molecular docking diagram of formononetin and three differentially expressed IRGs molecules. (a) IGF1. (b) ESR1. (c) CXCL12.

Molecular docking is an efficient auxiliary screening method to study the interaction between ligands and proteins and locate the best binding mode. Molecular docking has wide application prospects in the field of basic research on effective substances in traditional Chinese medicine. In this study, to further explore the interaction mechanism of formononetin with the four differentially expressed IRGs, a molecular docking model was constructed. IGF1 (PDB ID : 1GZR, binding energy: −6.05 kcal/mol), ESR1 (PDB ID : 1AKF, binding energy: −7.72 kcal/mol), and CXCL12 (PDB ID : 1SDF, binding energy: −6.77 kcal/mol) had a stable binding point with formononetin small molecular model, where the residue interacts via hydrogen bonds (Figure 7).

### 3.7. Correlation between IGF1, ESR1, CXCL12, and Immune Cell Infiltration in Breast Cancer

**Figure 8:**
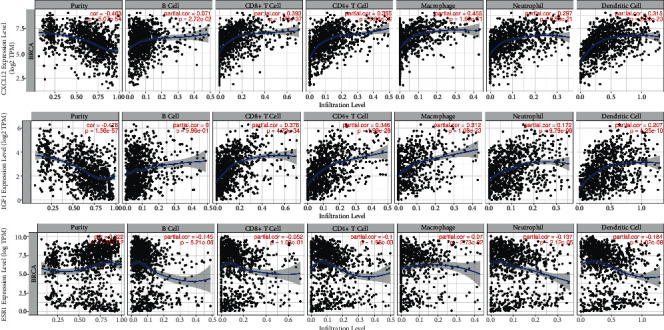
Correlation between three differentially expressed IRGs and immune cell infiltration.

The TIMER2.0 database was used to analyze the relationship between the three differentially expressed IRGs and immune cell infiltration in breast cancer. As shown in Figure 8, the abnormal expression of all three differentially expressed IRGs was correlated with massive infiltration of immune cells. The horizontal coordinates in the graph represent the abundance of immune cells and the vertical coordinates represent the gene expression levels, where *P* < 0.05 indicates a significant correlation between abnormal gene expression and immune cell infiltration.

## 4. Discussion

Breast cancer is among the most common and deadly malignancies found in women worldwide and is characterized by highly heterogeneous biological and clinical features [29]. The development of breast cancer is influenced by the interaction between cancer cells, the microenvironment, and the immune system [30]. In this study, GEO and ImmPort databases were used to identify key immune-related genes in breast cancer as novel therapeutic targets and to predict the key signaling pathways in which they are involved. Using bioinformatics analysis, we screened a total of 29 differentially expressed IRGs and performed GO and KEGG pathway enrichment analyses to identify key pathways in breast cancer immunity. The results revealed that differentially expressed IRGs in breast cancer were associated with PD-1/PD-L1-mediated immune escape, such as positive regulation of fibroblast proliferation, inflammatory response, positive regulation of ERK1 and ERK2 cascade, lymphocyte chemotaxis, estrogen signaling pathway, MAPK signaling pathway, NF-kappa B signaling pathway, Ras signaling pathway, and TNF signaling pathway.

PD-1 is a member of the B7-CD28 family which was discovered during activation-induced programmed cell death in a T cell hybridoma cell line [31]. The PD-1 gene is located on human chromosome 2q37, expressed as a monomer on the surface of the cell membrane, and involved in transmitting negative signals to activated T cells [32]. PD-1 is mainly expressed in activated CD4+ and CD8+ T cells, activated B cells, natural killer cells, natural killer T cells, dendritic cells, and activated monocytes [33]. Of the two ligands of PD-1—PD-L1 and PD-L2—PD-L1 is the main ligand, upregulated in many malignancies [34]. The interaction between both ligands on tumor cells with PD-1 on tumor-infiltrating lymphocytes (TILs) is considered as the main mechanism by which immune escape occurs in tumors [35]. The binding of PD-1 and PD-L1 in activated T cells induces tyrosine phosphorylation in the immunoreceptor tyrosine-based switch motif structural domain of PD-1 that in turn causes dephosphorylation of downstream protein kinases Syk and PI3K, inhibiting the activation of downstream AKT, RAS, ERK, and other pathways. This ultimately inhibits the transcription and translation of genes and cytokines required for T cell activation, negatively regulating T cell activity [36]. Cancer-associated fibroblasts (CAFs) play a key role in shaping the tumor immunosuppressive microenvironment in breast cancer. CAFs induce differentiation of recruited monocytes into M2-like macrophages, which are able to exert their immunosuppressive effects through the PD-1 axis [37].

Chinese herbal medicine and natural medicine—with their low toxicity and strong anticancer and chemoprotective properties—have long been used to treat tumors, either alone or as combination therapy [38]. Chinese herbal medicine and natural medicine contain multiple active ingredients that can act simultaneously on multiple targets, producing cumulative or synergistic effects [39]. Formononetin, a natural drug and the active component of the herb *Astragalus membranaceus*, can inhibit the migration and invasion of breast cancer cells by inhibiting MMP-2, MMP9, and PI3K/AKT signaling pathways [40]. Based on our results obtained using GEO disease data and molecular docking analysis, we suggest that the intervention of formononetin in breast cancer may be achieved by regulating IGF1, ESR1, and CXCL12.

Chemokine (C-X-C motif) ligand 12 (CXCL12) is a homeostatic chemokine secreted by fibroblasts, macrophages, and endothelial cells [41] and is highly expressed in different organs such as the liver, lungs, brain, lymph nodes, and bone marrow [42, 43]. CXC-chemokine receptor 4 (CXCR4) is the only known CXCL12 receptor, and the biological axis system formed by the two plays an important role in targeted metastasis of various malignancies including breast cancer, prostate cancer, hepatocellular carcinoma, and neuroblastoma [44, 45]. Breast cancer cells with high CXCR4 expression can metastasize to specific organs with CXCL12 chemotaxis, resulting in organ-specific metastasis. The CXCL12/CXCR4 axis also regulates cell proliferation, chemotaxis, migration, and adhesion by activating a series of intercellular signaling pathways and effectors [46, 47].

Insulin-like growth factor 1 (IGF1) is an important regulator of mammary gland development and tumorigenesis, enhancing mitosis, regulating the synthesis and secretion of hormones in the body, and influencing cell chemotaxis, immunity, and migration. It not only acts on the stroma of breast cells but also stimulates estrogenic activity in breast cancer patients and promotes ovarian excitation, thereby increasing the estrogen levels in the body. IGF1 synergizes with estrogen to promote the proliferation of breast cancer cells and increase their metastatic potential [48]. IGF1 binds to tyrosine kinase membrane receptor IGF1R, activates tyrosine kinase, recruits and phosphorylates insulin receptor substrate proteins, and activates intracellular signaling pathways involved in a variety of cellular activities, including proliferation, apoptosis, and migration [49, 50]. Studies have shown that formononetin can inhibit the proliferation of MCF-7 cells by inactivating the IGF1/IGF1R-PI3K/Akt signaling pathway [51]. Moreover, formononetin could reduce the expression of ESR1 in osteosarcoma and inhibit cell proliferation [52].

Estrogen receptor (ER), encoded by estrogen receptor 1 (ESR1), is a target for endocrine therapy. Alterations in the ESR1 like point mutations, gene amplification, and rearrangements cause conformational changes in the ER that can activate ER transcription function through a nonestrogen-dependent pathway and promote tumor cell growth [53, 54]. Chemokine (C-C motif) ligand 28 (CCL28) is secreted by epithelial cells. It is prominently found expressed in salivary, parotid, mammary glands, trachea, gastrointestinal tract, and prostate and to a lesser extent in leukocytes of peripheral blood and other mucosal tissues. CCL28 promotes breast cancer cell proliferation and metastasis through activation of the MAPK signaling pathway, upregulation of antiapoptotic protein Bcl-2, and inhibition of cell adhesion protein *β*-catenin. Moreover, it participates in the breast cancer progression and metastasis through ERK/MAPK-mediated antiapoptotic and metastatic signaling pathways [55]. The ER signaling pathway negatively regulates PD-L1 gene expression. In ER-negative breast cancers, high PD-L1 expression may lead to immune escape, while in ER-positive breast cancers, immune surveillance function may remain preserved due to low PD-L1 expression [56].

Currently, immunotherapy has been used to treat breast cancer via activating mechanisms of innate and adaptive immunities [57, 58]. Immunosuppression is closely related to the development of malignant tumors [59], making immunotherapy a promising treatment option for breast cancer. The various immunotherapies include engineered immune cells (CAR-T cell therapy), as well as checkpoint inhibitors (PD-1, PD-L1, and CTLA4) [60]. However, the majority of patients do not benefit these therapies, and up to 85% of patients are either innately resistant or have acquired resistance to immune checkpoint inhibitors [61]. If the patients do not derive the therapeutic benefit from immunotherapy, it not only delays treatment but also imposes a significant financial burden on them. Therefore, improving the efficacy of immune checkpoint inhibitors is crucial for effective immunotherapy of breast cancer.

Our study found a strong correlation between three differentially expressed IRGs and immune cell infiltration, and accumulating evidence suggests that they may interact with immune checkpoint inhibitors. Studies have demonstrated that CXCR4 inhibition promotes T cell accumulation and synergizes the antitumor effect of immune checkpoint inhibitors [62, 63]. Chen and colleagues reported that CXCR4 inhibition improved fibrous tissue proliferation, increased T-lymphocyte infiltration, and increased the effectiveness of immunotherapy in a metastatic breast cancer mouse model [64]. The results of several clinical studies have shown that the combination of CXCR4 antagonists and immune checkpoint inhibitors has good clinical results in patients with colorectal, pancreatic, and gastrointestinal cancers [65, 66]. IGF1R signaling negatively impacts immune surveillance in breast cancer patients, as evidenced by IGF1R phosphorylation resulting in slight CD8+ CTL infiltration, significant infiltration of FOXP3+ regulatory T cells, immunosuppressive CD163+ macrophages, and poor prognosis [67]. IGF1R and its downstream signaling molecules are susceptible to pharmacological inhibition by the immune system. Recent studies have shown that formononetin can inhibit PD-L1 expression by interfering with the interaction between MYC and STAT3, while enhancing the activity of CTLs and restoring their ability to kill tumor cells [68]. Therefore, formononetin, in combination with immune checkpoint inhibitors, is a promising therapeutic strategy in oncology.

Although our study identified possible pathological roles for IGF1, ESR1, and CXCL12 in breast cancer and a potential synergistic effect of formononetin with immune checkpoint inhibitors in breast cancer immunotherapy, the limitation of this study is that it is only a data analysis-based prediction. Further clinical and experimental validations are planned to explore the specific synergistic mechanism of formononetin with immune checkpoint inhibitors.

## 5. Conclusions

In conclusion, our findings predict that IGF1, ESR1, and CXCL12 may be effective targets of formononetin as a therapeutic for breast cancer. More importantly, formononetin might be synergistically combined with other therapeutics such as immune checkpoint inhibitors to increase the efficacy of immunotherapy by effective targeting of IGF1, ESR1, and CXCL12.

## Figures and Tables

**Table 1:** Top ten differentially expressed immune-related genes (IRGs) identified by topology analysis of the protein-protein interaction (PPI) network analysis.

No.	Gene	Degree	Betweenness	Closeness
1	CXCL12	14	186.85239	0.6756757
2	IGF1	13	134.64285	0.65789473
3	EGFR	11	94.86667	0.6097561
4	JUN	8	23.89762	0.5681818
5	CXCL2	8	15.726191	0.5102041
6	ESR1	7	26.288095	0.5555556
7	FOS	6	12.604762	0.5208333
8	SAA1	8	79.080956	0.5319149
9	CCL28	6	4.5833335	0.49019608
10	TGFBR2	6	30.180952	0.54347825

## Data Availability

The GSE103512 and GSE139038 data supporting this bioinformatic analysis are from previous datasets, which have been cited.
